# Are performance measurement systems useful? Perceptions from health care

**DOI:** 10.1186/s12913-017-2022-9

**Published:** 2017-01-31

**Authors:** Chiara Demartini, Sara Trucco

**Affiliations:** 1 Department of Economics and Management, University of Pavia, Via S. Felice, 5/7, 27100, Pavia, Italy; 2 Faculty of Economics, Rome University of International Studies, Via Cristoforo Colombo, 200, 00147 Rome, Italy

**Keywords:** Performance measures, Health care, Perceived managerial discretion, PLS-SEM, Process improvement

## Abstract

**Background:**

Prior literature identified the use of Performance Measurement Systems (PMS) as crucial in addressing improved processes of care. Moreover, a strategic use of PMS has been found to enhance quality, compared to non-strategic use, although a clear understanding of this linkage is still to be achieved. This paper deals with the test of direct and indirect models related to the link between the strategic use of PMS and the level of improved processes in health care organizations. Indirect models were mediated by the degree of perceived managerial discretion.

**Methods:**

A PLS analysis on a survey of 97 Italian managers working for health care organizations in the Lombardy region was conducted. The response rate was 77.6%.

**Results:**

The strategic use of PMS in health care organizations directly and significantly (*p* < 0.001) enhances performance in terms of improved processes. Perceived managerial discretion is positively and significantly (*p* < 0.001) affected by the strategic use of PMS, whereas the mediation effect is non-significant.

**Conclusions:**

This study contributes to the literature investigating the design and implementation of a non-financial measurement tool, such as the non-financial information included into a balanced scorecard (BSC), in health care organizations. Managers in health care organizations can benefit from the strategic use of PMS to effectively allocate their time to strategic opportunities and threats, which might arise and affect organizational, output-related performance, such as improving processes.

**Electronic supplementary material:**

The online version of this article (doi:10.1186/s12913-017-2022-9) contains supplementary material, which is available to authorized users.

## Background

One of the key questions regarding the use of PMS in the health care sector is: Are performance measurement system useful [1–4]? As Neely and his colleagues put forward, “a performance measurement system can be defined as the set of metrics used to quantify both the efficiency and effectiveness of actions” [5]. Recent national health policies, organizational practices and managerial effort have witnessed a change in the use of performance measurement tools within the health sector [6]. First, a macro-economic change refers to the use of performance measurement tools to detect and contain spending by health care organizations that generates deficit in the public sector. To do so specific recovery plans have to be agreed with either regional health authorities or the single organizations to regain efficiency within the health system [6]. Secondly, a change in the use of PMS occurs at the micro-economic level, whereby managers are called to collect, monitor and analyze information through PMS in order to improve the quality of care provided by health care organizations [7]. Thus, PMS are supposed to support managerial decision-making within health care organizations [8, 9], which, in turn, is expected to deliver improved and more efficient processes of care [10, 11]. Prior literature has identified the use of PMS as crucial in addressing improved processes of care [12]. Significant process improvements are process innovations, according to the Oslo Manual definition: “A process innovation is the implementation of a new or significantly improved production or delivery method. This includes significant changes in techniques, equipment and/or software” [13]. However, to date the literature on the use of PMS within health care organizations still requires more research [8, 14]. Hence, the effectiveness of PMS in delivering evidence-based care into practice is still open to debate [15, 16]. To bridge this gap, this paper aims at shedding some light on the relationship between the strategic use of PMS and the level of improved processes. Simons defined the strategic use of PMS as “the use of performance measurement system to detect strategic uncertainties. Strategic uncertainties relate to changes in competitive dynamics and internal competencies that may create opportunities or threats” [17]. As an example, an opportunity for health care organizations can be envisaged when two hospitals decide to merge to gain greater competitive power and increased efficiency. On the other hand, a negative report after an inspection can threaten the sustainability of a health care organization. A non-strategic use of PMS can be defined as a traditional feedback style of use of PMS to check for variances between target and actual performance [17]. For instance, budgetary control is often used in a non-strategic way by health care organizations to control hospital expenditures [18]. By grounding this paper on the upper echelon theory and perceived managerial discretion [19], this study assumes that the effectiveness of the strategic use of PMS on improved processes of care is mediated by the managerial discretion health care managers perceive in their area of responsibility [20]. Perceived managerial discretion refers to a manager’s perception of the “latitude of managerial action” [20]. Nonetheless, only a few studies have specifically addressed this topic in the public sector in general and in the health care sector in particular [8].

**Fig. 1:**
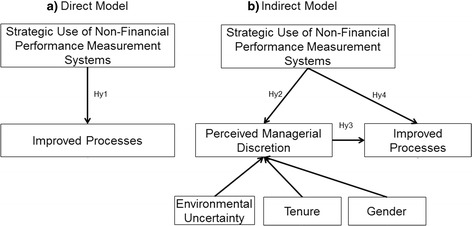
Conceptual framework. This file provides the theoretical framework of the research, by identifying the research hypotheses

Thus, this paper puts forward the following research questions: To what extent are PMS useful in enhancing improved processes of care delivery? Does the perceived managerial discretion mediate the relationship between the strategic use of PMS and improved processes of care? To test this conceptual framework this study uses data from a survey of 97 Italian (Lombardy region) health care managers. The conceptual framework is outlined in Fig. 1.

In doing so, this paper aims at contributing to the literature on the strategic use of PMS [21] and on the use of PMS in health care organizations [9]. This paper contributes also to the literature on perceived managerial discretion in several ways. First, results from this survey aim at replying to the call for more survey-based research on managerial discretion, in order to take into account for “many of the human factors that affect discretion” [22]. Second, this study puts forward a new antecedent of perceived managerial discretion, i.e., the strategic use of PMS.

The remainder of the paper is organized as follows. The next section will provide a review of the literature and the theoretical development of the conceptual framework. The third section will discuss research methodology, sample selection and constructs included in this study. The fourth section will analyze results from the surveyed sample. The fifth section will discuss empirical findings, practice implications, study limitations as well as future research avenues.

## Conceptual framework

### Characteristics of the Lombardy region health care system and its performance measurement system

The Lombardy region health service is one of the 21 regional health services in Italy. It accounts for 16.50% of the Italian population, with more than 10 million people living in Lombardy, and 16.39% of the total health budget, with a health expenditure of more than 17 billion euros [23]. It is based on three main pillars. First, a separation between local health authorities, which provide primary and community service, and hospitals, aimed at providing acute care. Secondly, the Lombardy region strongly supports patient’s choice of health care providers. Thirdly, it is organized as a quasi-market [24], where providers can be either public or private. In the latter case, private providers can deliver national health service also [25]. This regional health system has always been conceived as a high quality one [12, 26], witnessed by the highest rate of attractiveness within the national system [27].

The monitoring health care performance is a fundamental objective at both the national and regional level [28]. Starting from 2010, the Italian Ministry of Health began measuring performance across and within regions regarding specific areas, that are quality, efficiency, and appropriateness [29]. In the following year, the national monitoring body, Agenas, introduced the Outcome Evaluation National Program (PNE; [30]) to benchmark and compare Italian hospitals on outcome measures related to short-term mortality, short-term readmissions, hospitalization for specific clinic conditions, surgical treatments, short-term complications after specific treatments and waiting lists. The dataset gathers data from all the hospitals in Italy on 45 different performance measures [31]. At the regional level, the Lombardy region started collecting data on a variety of social and health care dimensions. According to this data, Berta and colleagues analyzed hospital performance in the Lombardy region against quality of care, measured by mortality rates [12]. These findings contribute to the knowledge that managers need in order to effectively improve quality in a financially constrained health care system. Similarly, Macinati found evidence that subjective outcome measures are strongly correlated with the diffusion of quality management initiatives, i.e. process innovations, in the Italian setting [32].

However, these studies focus on the nature (outcome-based) of the measure only, without taking into account the role of the use (strategic v non-strategic) of such performance measures on process improvement. Moreover, within this performance measurement context, in order to achieve multiple objectives and successfully cope with a complex environment at the same time, more and more health care managers need to excel in decision-making [24]. To do so, health care PMS should be perceived as useful by managers in enhancing their decision-making capabilities and the role of perceived managerial discretion in improving processes in health care delivery [33, 34]. As an example, Ozcan et al. take advantage of the value-based management approach to simulate and validate an optimization model for the efficient use of operating rooms in thyroid surgical wards [34]. Financial-, activity- and outcome-based performance are used by managers in order to effectively make decisions regarding the allocation of resources (operating rooms), thus improving processes and maximizing hospital performance [34]. Moreover, Elg and colleagues identified six activities where PMS improves decision–making and performance in clinical departments, i.e. continuous follow-up in formal arenas and meetings; improvement work; professional efforts; goal deployment; reporting based on external demands; and creating awareness in everyday clinical work [4].

### Strategic use of non-financial PMS, process improvements and perceived managerial discretion

Process improvements are pivotal to quality enhancement in the health sector. To improve processes, Simons pointed out that managers are encouraged to use PMS to scan their environment and detect strategic opportunities or threats in order to choose the most suitable options, i.e. a strategic use of PMS [21]. This is consistent with Grigoroudis et al.’s work, where the hospital they analyzed shows some weaknesses with regard to the performance in the customer perspective. However, a strategic use of the PMS enabled the management to “plan specific improvement actions in the next periods”, such as the redesign of the diagnostic services to improve the customer perspective [8, 9]. Prior literature found mixed results on the relationship between strategic use of non-financial PMS and process improvement. Some scholars found positive effects [8, 9, 35–37]. Indeed, theoretical and empirical results from the non-financial performance measurement literature have stressed that the non-financial information reported in the balanced scorecard (BSC) and other non-financial performance measurement tools improved quality of the health care service [8, 38]. Naranjo-Gil and Hartmann found that CEOs of hospitals should use PMS strategically to improve the quality of the service delivered [39]. Moreover, prior studies have stressed the need for health care managers to use non-financial performance indicators in a strategic way in order to align operational activities with organizational strategy [37], facilitate strategic change [9], enhance decision-making, and address strategic benchmarking to improve organizational innovation performance [40, 41]. In the heath sector the non-financial PMS have been found to effectively support forward-looking, and innovation-oriented decisions [36].

On the other hand, there are also studies reporting unintended consequences in the use of PMS, such as a lack of supportive environment for quality and process improvements, and the difficulty in using data to support change [42].

Therefore, more research aimed at disentangling the cause-effect relationship is needed [8]. In order to shed some light on the effect of the strategic use of non-financial PMS on the level of improved processes, this paper aims at testing the following direct relationship.

#### Hypothesis 1

A strategic (opposed to non-strategic) use of non-financial PMS positively impacts the level of improved processes.

The role of PMS in driving enhanced decision-making is quite controversial. On the one hand, health care staff members perceive performance measurement as a constraining tool to their decision-making autonomy [8]. In such a setting, the use of PMS could be misleading, i.e., grounded on a ‘hitting the target, but missing the point’ culture [43]. On the other hand, prior studies stressed that PMSs support quality process improvements, since they provide evidence-based metrics, which help to overcome quality issues in service delivery [8].

Starting from these assumptions, this paper analyses the concept of perceived managerial discretion [20], which is supposed to affect the degree of decision-making effectiveness [44, 45]. This study argues that the perceived managerial discretion is affected by the strategic use of non-financial PMS aimed at identifying strategic opportunities or threats. This paper focuses on non-financial measures rather than financial ones, since any kind of organizations adopts almost the same financial measures [46]; whereas non-financial performance measures must be organization specific to effectively support the achievement of organizational objectives [47], especially in the health care sector [39, 48, 49]. Artz et al. found that the use of specific and customized metrics, such as in the case of non-financial performance measures, has a positive effect on decision making and decision influence [50]. This is particularly true in the Italian NHS, where Ferrè et al. found that some of the most important challenges for the Italian NHS are (a) to develop a comprehensive database of quantitative and qualitative information and (b) to effectively align national and local managers’ discretion, in order to effectively implement health care reforms [6]. In other national contexts, similar considerations can apply. For instance, in the English National Health Service, the discretion of professionals is supported by a more strategic use of performance metrics, routines and guidelines [44]. In order to explore further the relationship between the use of non-financial PMS and perceived managerial discretion, this paper will test the following Hypothesis.

#### Hypothesis 2

A strategic (opposed to non-strategic) use of the non-financial PMS positively affects the perceived managerial discretion.

### Perceived managerial discretion and process improvement

The literature on perceived managerial discretion has mainly focused on determinants of perceptions, rather than on consequences. However, a stream of research has identified some of the main consequences of perceived managerial discretion. For instance, Phillips et al. [45] argued that managerial discretion and stakeholder orientation stimulates a feedback loop of dynamic managerial discretion. Their assumptions have not been tested yet. Carpenter and Golden tested the relationship between perceived managerial discretion and managerial power, defined as the “ability to influence others” [44]. A simulation involving twenty managers found support only for their hypothesis on the relationship between low discretion and managerial power.

The health care literature failed to effectively show consequences of the perceived managerial discretion. On the one hand, some studies have focused primarily on clinical consequences, such as the number of treatment errors and perceived staff discretion [51]. On the other hand, others have identified more financial-related consequences [45]. The former stream of literature is more related to quality improvements, whereas the latter is mainly concerned with efficiency targets. The literature on the relationship between perceived managerial discretion and improved organizational processes is quite fragmentary also. By surveying 87 German hospitals Schultz and his colleagues provided empirical support to the hypothesis that a stronger (weaker) analytical orientation, based on informed decision-making, addresses a higher (lower) level of innovativeness [10]. Prior studies have addressed the opposite relationship, i.e., the effect of process innovation on managerial discretion [52]. In his study, Hoff posited that two process innovations in primary care, namely the use of electronic medical records and the introduction of clinical practice guidelines, actually resulted in de-skilling outcomes, measured by (a) decreased clinical knowledge, (b) decreased trust with patients, (c) “one-size-fits-all patient”, (d) decreased decision-making confidence, and (e) inaccurate patient information. Since there is a call for more research on “the role that healthcare managers can play” in innovation implementation [52] and diffusion [53], this study aims to investigate the following:

#### Hypothesis 3

Perceived managerial discretion has a positive effect on the level of improved processes.

In order to check for mediating effects on the link between the strategic use of non-financial PMS and improved processes, this study grounded the conceptual analysis on contingency theory, which tests the fit between the design/use of PMS and contingency variables [35]. Actually managerial capabilities and other soft skills enable health care managers to effectively interpret non-financial performance and translate it into a decision-making process aimed at both identifying and selecting those innovations that will result in enhanced processes [36]. According to this conceptual development, this paper contends that the role of perceived managerial discretion mediates the relationship addressed in Hypothesis 1. Following these arguments, this paper proposes the following:

#### Hypothesis 4

Perceived managerial discretion fully mediates the relationship between the strategic use of non-financial PMS and the level of improved processes.

## Methods

### Sample selection and data collection

To test the research hypotheses, this study was based on an empirical study on a sample of Italian managers in hospital structures located in the Lombardy region (Northern Italy). The choice for this region is due to its efficiency in the resource management process and in the overall PMS [6]. The responsibility center is the organizational unit of analysis. A responsibility center is the organizational unit, however named, that makes the decisions regarding both the use of resources and innovation [36]. As a preliminary step, the total amount of organizational units has been identified, which is assumed to be around 2,000, determined by multiplying the number of hospitals in Lombardy (200 structures according to [54]) and the amount of responsibility centers per structure in Lombardy region (10 on average). To collect data, this paper adopted an approach based on an anonymous paper questionnaire sent to 125 responsibility centers, who have both clinical and administrative responsibilities. A random sampling selection method was performed to identify the final 125 units of analysis among the universe of units, since it is a common practice in defining the sample in a survey in the health care sector [55, 56] and in this way, all participants have an equal chance of selection [57].

The final sample can be considered representative of the whole population, since in our sample each feature of the organizational units is taken into consideration; for example public and private units, teaching and research units (see Table 1). Therefore, we tested the mean differences in key features used to identify the sample that are: 1) private not-for-profit and public entities; 2) teaching research and non-research centers; 3) number of beds and; 4) managerial tenure between the overall population and the subset of respondents [58]. Results for non-response bias highlighted no concerns about non-response bias and this allows us to confirm that the final sample was representative of the entire population. The source of our data for non-response analysis is the statistical dataset of the Lombardy Region and the Italian Ministry of Health, Statistical department.

**Table 1:** Demographic statistics of the sample under analysis

		Hospitals	Sample managers
Type of hospitals	Private	2	23
	Public	5	102
	Total	7	125
Complexity of hospitals	Research	3	38
	Teaching	4	87
	Total	7	125
Avg number of beds		852.99	23
Avg catchment area		502,352	27,840
Gender	Male	90	101
	Female	35	24
	Total	125	125

In order to determine the sample size which is representative, we applied the following formula [59]:

$$ \mathrm{n}=\left({\mathrm{F}}^2\kern0.5em \times \kern0.5em \mathrm{N}\kern0.5em \times \kern0.5em \left(\mathrm{P}\kern0.5em \times \kern0.5em \left(1{\textstyle \hbox{-}}\mathrm{P}\right)\right)\right)/\left(\left(\mathrm{D}{\mathrm{S}}^2\kern0.5em \times \kern0.5em \left(\mathrm{N}{\textstyle \hbox{-} }1\right)\right)+\left({\mathrm{F}}^2\kern0.5em \times \kern0.5em \mathrm{P}\kern0.5em \times \kern0.5em \left(1{\textstyle \hbox{-}}\mathrm{P}\right)\right)\right) $$

Where desired precision (DS) = 2%; positive results with probability *P* = 99%; degree of confidence = 95% (F = 2); population of manager = N. By applying this formula, we found that *n* = 94 [60].

Furthermore, since a PLS – SEM analysis was performed, some scholars recommend that the sample size should fulfill both of the following requirements: 1) ten times the largest number of formative indicators for one construct (30 units of observation in this analysis); and 2) ten times the largest number of structural model paths [61] (60 units of observation in this analysis).

The questionnaire has been validated though a pilot study on both academics and clinicians with administrative roles for content and face validity [62]. Survey respondents could rely on a guide in the questionnaire with a clear definition of each concept used in this study in order to avoid misunderstanding of topics. We performed a single cross-sectional survey, since it is largely used in the health sector [63–65].

After two weeks from the survey submission, a reminder to all managers was sent. The response rate was 77.6% (97 valid questionnaires were returned), which can be considered in line with other similar studies in this sector and with the representative sample size [9].

A check for the distribution of the control question (How would you rate your overall satisfaction with the non-financial performance measurement system in use within your unit?) was performed through a plot analysis [66], and it can be assumed that it is in line with the distribution of the other research questions included into the questionnaire. Demographic statistics of the study sample are shown in Table 1.

To check for early and late respondents bias, a t-test analysis on early (managers who answered before the reminder) and late respondents (managers who answered after the reminder) was performed according to continuum of resistance model [67]. Results reject the hypothesis of bias between early and late respondents in this sample (Appendix). A follow-up procedure was also performed in order to convert refusers. This procedure has been found to reduce non-response bias [68].

### Variable measurement

Based on collected data and according to the research hypotheses, study variables were developed, namely: Strategic Use of Non-Financial Performance Measurement Systems, Perceived Managerial Discretion and Improved Processes. Prior literature has identified Tenure, Gender and Environmental Uncertainty as relevant control variables for both the perceived managerial discretion and improved processes within the health care management literature [10]. Thus, these variables were considered in this analysis too. Research variables, control variables, variables’ items and relative description are summarized in Table 2.

**Table 2:** Research variables, survey items and scale measurement (in %) for each variable (*N* = 97)

Research variable name	Survey questions addressed to health care managers and variable items	Variable measurement	Frequency distribution for survey questions (in %)							
			0	1	2	3	4	5	6	7
*Strategic Use of Non-Financial Performance Measurement Systems*	How would you rate the relevance in the use of non-financial PMS in detecting straegic uncertainties within your organizational unit?	A score from 1 to 7 on a Likert scale (1 Extremely unsatisfactory,…, 7 Extremely satisfactory)		2.062	10.309	14.433	29.897	21.649	17.526	4.124
*Perceived Managerial Discretion*	Decision Making: What is your perception about the effectiveness of the non-financial performance system used in providing information with reference to support operational decisions of your unit?	Decision making: a score from 1 to 7 on a Likert scale (1 Extremely unsatisfactory,…, 7 Extremely satisfactory)		2.062	6.186	11.34	11.34	19.588	39.175	10.309
	Flexibility: What is your perception on the effectiveness of the non-financial performance system used in providing information with reference to enabling the flexibility/adaptability of your organizational unit?	Flexibility: a score from 1 to 7 on a Likert scale (1 Extremely unsatisfactory,…, 7 Extremely satisfactory)		4.124	6.186	12.371	22.68	22.68	27.835	4.124
*Improved Processes*	Did your organizational unit introduce improved processes during the last three years?	A score from 1 to 7 on a Likert scale (1 Very much less than sector’s average,…, 7 Much greater than sector’s average). 0 if no improved processes have been introduced during the last three years.	18.557	2.062	5.155	9.278	14.433	28.866	18.557	3.093
*Tenure*	Time: How long have you been with the company?	Time: respondent has to indicate years and months (0 < "1" ≤ 5 years; 5 < "2" ≤ 10 years; 10 < "3" ≤ 15 years; 15 < "4" ≤ 20 years; 20 < "5" ≤ 25 years; 25 < "6" ≤ 30 years; "7" > 30 years)		40.206	7.216	5.155	7.216	17.526	8.247	10.309
	Time actual: How long have you been in the current job?	Time actual: respondent has to indicate years and months (0 < "1" ≤ 3 years; 3 < "2" ≤ 6 years; 6 < "3" ≤ 9 years; 9 < "4" ≤ 12 years; 12 < "5" ≤ 15 years; 15 < "6" ≤ 18 years; "7" > 18 years)		38.144	28.866	10.309	9.278	3.093	3.093	3.093
*Gender*	Women/Men managers	A dichotomous variable: 0 if respondent is a man and 1 if respondent is a woman.	79.381	20.619						
*Environmental Uncertainty*	Complexity: What is the level of complexity faced in your unit’s environment compared to the average of the sector you belong to?	Complexity: A score from 1 to 7 on a Likert scale (1 Very much less than sector’s average,…, 7 Much greater than sector’s average)		0	0	8.247	24.742	19.588	34.021	13.402
	Risk: What is the level of risk faced in your unit’s environment compared to the average of the sector you belong to?	Risk: A score from 1 to 7 on a Likert scale (1 Very much less than sector’s average,…, 7 Much greater than sector’s average)		1.031	4.124	15.464	27.835	30.928	17.526	3.093
	Uncertainty: What is the degree of uncertainty faced in your unit’s environment compared to the average of the sector you belong to?	Uncertainty: A score from 1 to 7 on a Likert scale (1 Very much less than sector’s average,…, 7 Much greater than sector’s average)		2.062	8.247	7.216	42.268	27.835	10.309	2.062

The Strategic Use of Non-Financial Performance Measurement Systems variable highlights the strategic use of non-financial performance measures [21]. This variable ranges in a continuum from non-strategic use to strategic use of non-financial performance measurement system, with low scores associated to low strategic use (i.e. non-strategic use) and higher scores linked to high strategic use of PMS. With reference to the Perceived Managerial Discretion variable, this study tried to overcome the limitations of prior studies by using a measure, which directly assesses this perception and is not a proxy for it [51]. Specifically, this variable represents the mean value of the scores assigned to Decision-Making and Flexibility by respondents. The former dimension – perceived support to decision-making – refers to prior literature on perceived managerial discretion, which has pointed out that the perception of the latitude of options available to managers is closely related to strategic decision-making [44]. The latter dimension – perceived flexibility – is consistent with previous studies, such as Kogut and Kulatilaka’s [69] in which managers perceive operational flexibility as an inhibiting/enabling factor for executives’ perceived discretion. As stated by Simons, organizations demand both innovation and flexibility. Flexibility entails a manager’s autonomy, freedom and control over the actions under their responsibility [15]. Improved Processes represents a variable of process innovation (new or significantly improved methods for the production or supply of products; [35]). Tenure variable is the sum of the manager’s tenure in the same company and in the current position. Finally, Environmental Uncertainty represents the mean of Complexity, Risk and Uncertainty based on other studies [70]. Each ordinal and nominal variable was standardized in order to avoid problems involving the heterogeneity of data.

### Research model

To test the research hypotheses, a Partial Least Squares-Structural Equation Modeling (PLS-SEM) was performed on the whole dataset of answers by the 97 managers. A direct PLS analysis and an indirect one was performed by using the SmartPLS software package [71]. PLS-SEM is a causal modeling approach aimed at maximizing the explained variance of the endogenous latent variables widely used across disciplines such as marketing [72], strategic management [73], and health care [74]. Prior studies that use PLS-SEM have shown the benefits of this statistical approach, i.e. effectiveness in the application to small sample sizes, non-normal data, formative measures of latent variables, complex relationships with multiple dependent variables, issues with a scarcity of prior theoretical literature and in measuring the reflective relations between research constructs and variables which are believed to reflect the unobserved construct [75] (e.g., [71]).

According to literature recommendations, the PLS-SEM was performed to provide estimates of the relations between variables and constructs (measurement model) and among constructs (structural model) [71]. The PLS-SEM method consists of two main steps: 1) the analysis of the reliability and validity of the measurement model, and 2) the analysis of the structural model [73]. Since PLS path modeling does not provide a goodness-of-fit criterion, the study drew on Chin’s study [75] to assess partial model structure. In order to assess the measurement model, this paper tested (a) the internal consistency reliability, (b) the convergent and discriminant validity for the latent variables, (c) the factor loading for each indicator included in the latent variable, and (d) the cross validated redundancy [72]. Cronbach’s alpha and composite reliability values of all the latent constructs achieve satisfactory levels (α > 0.7) for early stage research studies (Table 3). To validate the reliability of formative variables, Harman’s single factor test for common method variance was performed for Perceived Managerial Discretion, for Environment Uncertainty and for Tenure. This test shows the presence of single factor for the above-mentioned variables [76]. Convergent validity has been assessed by the average variance extracted (AVE; [75]). Each construct achieved a level of validity well above the satisfactory 0.5 threshold [75] (Table 3). Discriminant validity was checked by running a cross-loadings procedure [75]. To assess each indicator’s weights significance a bootstrapping test was performed [75]. As shown in Table 3, indicators included in the latent variables present a satisfactory level of statistical significance. Cross-validated communality values are positive for all latent variables included in the model, therefore allowing us to state that the quality of the measurement model is good [72].

**Table 3:** Descriptive statistics of the research variables, reliability and validity of constructs, and the goodness-of-fit of the structural model, correlation matrix and Pearson index

Variable	Number	Factor Loading (p-value)	Min	Max	Mean	Standard Deviation	AVE (Average Variance Extracted)	Composite Reliability	Chronbach's Alpha	CVR (Cross-Validated Redundancy)	Effect size (f^2^)	Perceived Managerial Discretion Pearson (Sig two-tailed)	Improved Processes Pearson (Sig two-tailed)	Strategic Use of Non-Financial Performance Measurement Systems Pearson(Sig two-tailed)	Tenure Pearson (Sig two-tailed)	Gender Pearson (Sig two-tailed)	Environmental Uncertainty Pearson (Sig two-tailed)
*Perceived Managerial Discretion*	97		1	7	4.763	1.429	0.914	0.955	0.906	0.301	0.017	1					
*Decision Making*		0.955 (^a^)	1	7	4.990	1.510											
*Flexibility*		0.957 (^a^)	1	7	4.540	1.479											
*Improved Processes*	97		0	7	3.760	2.168				0.050		0.254; (0.012^b^)	1				
*Strategic Use of Non-Financial Performance Measurement Systems*	97		1	7	4.280	1.405				0	0.381	0.581; (0.000^c^)	0.121; (0.250)	1			
*Tenure*	92		0	55	17.804	14.430	0.781	0.876	0.727	0	0.022	0.238; (0.022^b^)	0.121; (0.250)	0.222; (0.033^b^)	1		
*Time*		0.925 (^a^)	0	37	13.043	11.222											
*Time Actual*		0.840 (^a^)	0	20	4.7310	4.658											
*Gender*	94		0	1	0.210	0.411				0	0.003	0.138; (0.186)	0.032; (0.757)	0.145; (0.164)	0.223; (0.034^b^)	1	
*Environmental Uncertainty*	97		2	7	4.643	0.952	0.612	0.823	0.702	0	0.021	0.176; (0.085)	0.082; (0.422)	0.143; (0.163)	−0.198; (0.059)	−0.131; (0.209)	1
*Complexity*		0.823 (^a^)	3	7	5.200	1.196											
*Risk*		0.868 (^a^)	1	7	4.480	1.217											
*Uncertainty*		0.638 (^a^)	1	7	4.250	1.191											

^a^Statistically significant at the *p* < 0.001 level. ^b^Correlation is significant at 0.05 (2-tails). ^c^Correlation is significant at 0.01 (2-tails)

To assess the structural model the following tests were checked: (a) R^2^ of endogenous latent variables, (b) estimates for path coefficients, and (c) cross-validated redundancy (*f* ^2^, [72]). The R^2^ of Perceived Managerial Discretion shows a moderate level, while Improved Processes presents a weak coefficient of determination [75] (Table 4). Moderate levels of R^2^ can be accepted when the number of exogenous latent variables explaining the endogenous latent variable is low, as in this research model [72]. This path model shows path coefficients whose sign is aligned to the theoretical assumptions, i.e., they are all positive. The magnitude and significance of the path coefficients achieve satisfactory levels (p-value < 0.01; Table 4). Cross-validated redundancy values for all exogenous variables are positive (CVR, Table 3). Thus, the structural model shows a satisfactory quality level [72].

**Table 4:** Regression weights for the PLS-SEM analysis

	Direct Model	Indirect Model	
	(a)	(b)	
	*n* = 97	*n* = 97	
Variable (Y)	R square	R square	
Perceived Managerial Discretion		36.30%	
Improved Processes	6.80%	8.40%	
	*Path coefficient*	*Direct Path Coefficient*	*Indirect Path Coefficient*
	(*P*-value)	(*P*-value)	(*P*-value)
*Perceived Managerial Discretion- > Improved Processes*		0.154	
		(0.138)	
*Strategic Use of Non-Financial Performance Measurement Systems - > Perceived Managerial Discretion*		0.524	
		(0.000^a^)	
*Strategic Use of Non-Financial Performance Measurement Systems - > Improved processes*	0.261	0.171	0.081
	(0.008^a^)	(0.118)	(0.189)
*Tenure- > Perceived Managerial Discretion*		0.129	
		(0.136)	
*Gender- > Perceived Managerial Discretion*		0.045	
		(0.579)	
*Environmental Uncertainty- > Perceived Managerial Discretion*		0.121	
		(0.257)	
*Environmental Uncertainty- > Improved Processes*			0.019
			(0.437)
*Gender- > Improved Processes*			0.007
			(0.657)
*Tenure- > Improved Processes*			0.020
			(0.350)

^a^Statistically significant at the *p* < 0.001 level (two-tailed; bias-corrected bootstrap confidence interval)

Table 3 shows some descriptive statistics of the research variables.

## Result

**Fig. 2:**
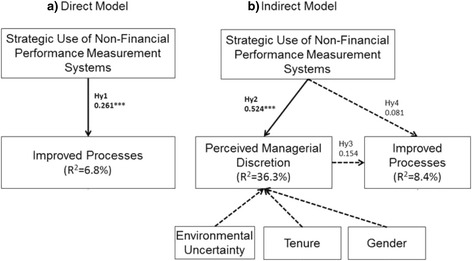
Path analysis results. This file provides path results of the research

Results from the SEM-PLS analysis on the study sample are summarized in Table 4 and in Fig. 2. Hypothesis 1 and Hypothesis 4 test both the direct model – related to the effect of the Strategic Use of Non-Financial PMS on the amount of Improved Processes (Hy 1) – and the mediated model, where the Perceived Managerial Discretion mediates the direct model (Hy 4). Analysis from the direct model provides a positive and statistically significant path coefficient (at the *p* < 0.001 level). Thus, the greater the Strategic Use of Non-Financial PMS is, the higher the level of Improved Processes. Although the direct path coefficient (0.171) and indirect one (0.081) are positive in the indirect model too, the level of statistical significance is very low (*p* > 0.1). In this case, the coefficients of determination in both the direct and indirect models that refer to Improved Processes are quite low (direct model: R^2^ = 6.8%; indirect model: R^2^ = 8.4). Even though the Hypothesis 4 cannot be supported by empirical findings, the paper provided new insights regarding the relationship between the strategic use of PMS and innovation. In particular, we can extend results from Bisbe and Otley’s study [77]. In their study, they couldn’t find any significant mediated relationship between the strategic use of PMS and innovation. Thus, they called for further research in this field. In this study, we demonstrated that the lack of significant results could be due to a model misspecification. By introducing the moderating term – perceived managerial discretion – in the direct model, we enhanced the coefficient of determination of the relationship between strategic use of PMS and process innovation. Besides the research variable “perceived managerial discretion” is able to increase value in the coefficient of determination of the link between the strategic use of PMS and perceived managerial discretion, since the R^2^ is quite moderate (36.3%). Therefore, more investigation on the mediated effects between strategic use of PMS and process innovation is needed in order to identify other determinants that could have some effects along with the perceived managerial discretion.

The dataset provided empirical support to Hypothesis 2, which tested the direct effect of a Strategic Use of Non-Financial PMS on the degree of Perceived Managerial Discretion. In fact, the path coefficient for this relationship is positive and significant (at *p* < 0.001). In addition, the explanatory power of the SEM-PLS model is moderate (R^2^ = 36.3%), as previously stated, but provides large room for future research. As regards Hypothesis 3, testing the direct effect of Perceived Managerial Discretion on Improved Processes, the empirical findings are in line with the theoretical assumptions: a higher perception of managerial discretion positively impacts the level of improved processes. Nonetheless, the degree of statistical significance is very low (*p* > 0.1). Thus, more investigation is needed in order to shed some light on this relationship.

The three control variables, that is environmental uncertainty, tenure and gender, don’t provide a significant effect on perceived managerial discretion. This enables us to state that the contingency effect of the three control variables on perceived managerial discretion is not relevant in this study.

## Discussion

This study contributes to the literature investigating the design and implementation of a non-financial measurement tool, such as the non-financial information included into a balanced scorecard (BSC), in health care organizations [8]. In fact, until now studies on the use of BSC within the health care context have been primarily aimed at analyzing the design and implementation of this managerial tool, rather than the effect of its use on relevant outcome variables [9]. In this sense, this paper’s aims is to contribute to the literature on the strategic use of PMS [21] and the use of PMS in health care organizations [36]. This is particularly relevant within the health care sector, where the need for managerial ambidexterity [78] - in terms of both efficiency in resource usage and innovation in exploitation of new pathways of care – should be effectively fulfilled by a strategic use of PMS [21]. Thus, this paper analyzed the mediating role of perceived managerial discretion in the aforementioned relationship. However, we can extend results from Bisbe and Otley’s study [77]. In their study, they couldn’t find a significant mediated relationship between the strategic use of PMS and innovation. Thus, they called for further research in this field. In this study, we demonstrated that the lack of significant results could be due to a model misspecification. By introducing the moderating term – perceived managerial discretion – in the direct model, we enhanced the coefficient of determination of the relationship between strategic use of PMS and process innovation. Therefore, more investigation on the mediated effects between strategic use of PMS and process innovation is needed in order to identify other determinants that could have some effects along with the perceived managerial discretion.

As a matter of fact, empirical findings from this study support Hypothesis 2, according to which the strategic use of non-financial PMS has a positive and direct effect on the perceived managerial discretion. These findings are consistent with two streams of studies based on the upper echelon theory. First, the cognitive theory of attention and bounded rationality [21], which has been integrated with the upper echelon theory in Cho and Hambrick’s study [79]. They demonstrated that managers – whose time is a scarce resource – can effectively benefit from the use of PMS in order to effectively allocate their time to detect and manage strategic opportunities and threats, which might arise and affect organizational performance. Prior studies on the prioritization of decisions put forward that senior managers know which are the strategic decisions, but they fall short in effectively allocating their time to the analysis and implementation of those decisions [80]. In doing so, we extended Cho and Hambrick’s analysis by replying to the research question they addressed in order to investigate further the relationship between upper echelon theory and attention theory, that is “what are the factors that engenders attention?” [79]. We demonstrated that the strategic use of non-financial PMS is a key factor in both enabling prioritization of objectives and focusing managerial attention. In the health sector, prioritization of decisions is crucial since it involves at least two dimensions, the financial one, attached to the efficient use of public resources [81], and the outcome-related one, which has more to do with population health and quality of care [82]. Second, in line with prior research [9] this paper found that the strategic use of non-financial PMS fosters managers’ capability to frame the set of options they can put in place in order to excel in decision-making, i.e. to extend their perceived managerial discretion. In his studies Naranjo-Gil obtained similar evidence on the influence of managerial features on the style of use of the BSC from a sample of Spanish nurse managers [9] and hospital managers [2]. These findings corroborate further the upper echelon theory, according to which managers’ behavior is predicted by their personal characteristics and background [19, 20, 39, 44]. More specifically, it addresses the attitude to the strategic use of PMS as a key feature in perceiving an extended managerial discretion. Indeed, social psychological theories address the need for managers to perceive to be able to cope with situational constraints and situational strengths [51]. According to self-determination theory, managers perceiving more autonomy will also be more motivated to perform [45]. Thus, a more strategic, rather than non-strategic, use of PMS might result in more empowered health care managers. Hence, this paper aimed at replying to the call for more research on the use of PMS to improve effectiveness-oriented measures, such as the perception of managerial discretion, in health care organizations [83].

## Conclusions

This study aimed at analyzing whether the strategic use of non-financial PMS directly enhances the level of improved processes in a health care setting. Moreover, according to the upper echelon theory, this paper investigated whether the perceived managerial discretion mediates the prior relationship. Empirical findings support theoretical arguments on the direct and positive relationship between the strategic use of management accounting tools and the level of improved processes in health care. Results from the PLS-SEM analysis on the research dataset do not support the mediation of perceived managerial discretion on the link between strategic use of PMS and innovation.

From a methodological standpoint, this research contributed to the measurement of perceived managerial discretion [84]. This paper put forward a subjective measure, which achieved a satisfactory level of internal consistency and validity. Although this measure is not based on psychological theories, such as in prior studies [45], this paper tried to overcome limitations of prior measurement approaches by using a measure which directly assesses the perception of managerial discretion while not being a proxy of it [51].

From a public policy perspective, health regulators can use empirical evidence from this study in order to spread knowledge to health providers on the effects of different uses of PMS to enhance quality in the health sector.

This study is not without its limitations. The results reported in this study refer to a sample of managers operating in the Italian Lombardy region NHS; thus, caution should be used in generalizing such findings. Furthermore, future research could be addressed at extending the sample to managers of other Italian regions and other countries in order to facilitate comparisons among different NHS systems by taking into account different cultural settings too [70]. The extension of the sample could allow researchers to apply other statistical methods than the PLS-SEM one. Empirical findings regarding the relationship between Perceived Managerial Discretion and Improved Processes (Hy 2) and the mediating effect of Perceived Managerial Discretion on the relationship between Strategic Use of Non-Financial Performance Measurement Systems and Improved Processes (Hy 4) are aligned to the theoretical expectations. However, the statistical significance is too low, thus additional research should also be addressed at investigating these links.

We have measured complex constructs with survey items, therefore a more rigorous approach could be carried out to develop and validate survey scales on these constructs. Future research could be addressed to validate this survey’s items to build our constructs and to provide more useful items able to explain the research constructs. Since the questionnaire was single cross-sectional, it is difficult to infer any causality among research variables, therefore further studies to evaluate the causality among features are strongly encouraged. Moreover, some measures used in this study are based on managerial perceptions. Although these measures might induce some bias from self-reported information, this paper asked respondents to compare their perception of discretion with the average of their sector, in order to reduce this measurement error. Further research could also compare and contrast results from this construct on perceived managerial discretion with others already available within the literature.

## Appendix

Appendix provides early and late response analysis.

### Early and late response analysis

A test on an early-late response has been conducted for all the relevant research variables (Strategic Use of Non-Financial Performance Measurement Systems; Perceived Managerial Discretion; Improved Processes; Environmental Uncertainty) to check for differences in the two groups following a wave analysis proposed by Rogelberg and Stanton [86]. Results, obtained performed a Two-sample t test with equal variances, show that the mean differences of the variables included in the research models are not statistically significant; which again enable us to reject the hypothesis of bias between early and late respondents in surveyed sample (Strategic Use of Non-Financial Performance Measurement Systems: t = 1.754; Probability = 0.083; Perceived Managerial Discretion: t = 0.899; Probability = 0.371; Improved Processes: t = 1.641; Probability = 0.104; Environmental Uncertainty: t = 0.760; Probability = 0.449). This approach is consistent with this study’s methodological choices to focus on responsibility centers as units of analysis. Furthermore, this approach is regarded as valid concerning the sampling strategy in mail survey method and thus has been adopted in a lot of published research (e.g. [77]). Therefore, we tested the mean differences in key features used to identify the sample (private non for profit and public entities, research and non-research centers; number of beds and managerial tenure) between the overall population and the subset of respondents [87]. These key features address the main characteristics of the Italian NHS with particular regard to the Lombardy Region, whose population represents 16.75% of the Italian population (Italian Minister of Health care, Statistical Department). We checked for differences in the type of the responsibility centers (whether private non for profit/public) since there may be some differences in the features of the performance management system in use due to different regulations and the degree of organizational autonomy [88]. In the analyzed dataset, 11 out of 97 (11.34%) of responsibility centers are private non for profit entities. We also checked for differences in another typology of the responsibility centers (whether teaching and research or non-teaching units), because prior literature pointed out that teaching and research centers have a high informational needs regarding performance management system compared to other health care organizations [83]. In this study dataset, 16 out of 97 (16.49%) of responsibility centers are teaching and research units. We checked for differences in number of beds, since the complexity of the unit of analysis affects the managerial need for information with specific regard to performance management system [89]. In the surveyed dataset, each responsibility center has 91 beds on average. Finally, we checked for differences in managerial tenure, since the managerial tenure of the unit of analysis affects the performance management system [10]. In this study dataset, each responsibility center has on average a managerial tenure of 13.04 years.

Results for non-response bias on the private non for profit/ public entity are based on Kruskal-Wallis equality-of-populations rank test, since the variable is a dichotomous one [90, 91] and highlighted no concerns about non-response bias (chi-squared = 0.813; probability = 0.367). Similar conclusions can be drawn from the non-response bias analysis on the teaching and research/non-research centers. Kruskal-Wallis equality-of-populations rank test for dichotomous variable highlighted no concerns about non-response bias (chi-squared = 3.154; probability = 0.076).

Also another non-response analysis for number of beds revealed no concerns about non-response bias. We performed a two-sample t test with equal variances, since this variable is an ordinal one [87]. Results of this test revealed no concerns about non-response bias (t = 1.407; Probability = 0.161). The source of data for non-response analysis is the statistical dataset of the Lombardy Region (https://www.dati.lombardia.it/browse?category=Sanit%C3%A0&sortBy=relevance&utf8=%E2%9C%93).

Furthermore, the last non-response test we checked for, that is managerial tenure, revealed no concerns about non-response bias. We performed a two-sample t test with equal variances, since this variable is an ordinal one (t = -0.5329; Probability = 0.5946). The source of data for managerial tenure is the Italian Minister of Health, Statistical department (http://www.salute.gov.it/imgs/C_17_pubblicazioni_1816_allegato.pdf).

These results along with the non-response analysis findings strongly assure that this study results are not biased.

## Additional files

Additional file 1: Questionnaire. This file provides information regarding the questions included into the survey related to this study. (DOCX 1713 kb)
Additional file 2: Database. (XLS 39 kb)

## Abbreviations

BSC: Balanced scorecard
NHS: National health service
PLS-SEM: Partial least squares-structural equation modeling
PMS: Performance measurement systems

## References

[1] Amaratunga D, Haigh R, Sarshar M, Baldry D (2002). Application of the balanced score‐card concept to develop a conceptual framework to measure facilities management performance within NHS facilities. Int J Health Care Qual Assur.

[2] Adcroft A, Willis R (2005). The (un)intended outcome of public sector performance measurement. Int J Public Sect Manag.

[3] Valderas JM, Fitzpatrick R, Roland M (2012). Using health status to measure NHS performance: another step into the dark for the health reform in England. BMJ Qual Saf.

[4] Elg M, Palmberg Broryd K, Kollberg B (2013). Performance measurement to drive improvements in healthcare practice. Int J Oper Prod Manag.

[5] Neely A, Mills J, Platts K, Huw R, Gregory M, Bourne M (2000). Performance measurement system design: developing and testing a process‐based approach. Int J Oper Prod Manag.

[6] Ferrè F, Cuccurullo C, Lega F (2012). The challenge and the future of health care turnaround plans: evidence from the Italian experience. Health Policy Amst Neth.

[7] Trebble TM, Paul M, Hockey PM, Heyworth N, Humphrey R, Powell T (2015). Clinically led performance management in secondary healthcare: evaluating the attitudes of medical and non-clinical managers. BMJ Qual Saf.

[8] Grigoroudis E, Orfanoudaki E, Zopounidis C (2012). Strategic performance measurement in a healthcare organisation: A multiple criteria approach based on balanced scorecard. Omega.

[9] Naranjo-Gil D (2009). Strategic performance in hospitals: The use of the balanced scorecard by nurse managers. Health Care Manage Rev.

[10] Schultz C, Zippel-Schultz B, Salomo S (2012). Hospital innovation portfolios: Key determinants of size and innovativeness. Health Care Manage Rev.

[11] Varabyova Y, Blankart CR, Torbica A, Schreyögg J (2016). Comparing the Efficiency of Hospitals in Italy and Germany: Nonparametric Conditional Approach Based on Partial Frontier. Health Care Manag Sci.

[12] Berta P, Seghieri C, Vittadini G (2013). Comparing health outcomes among hospitals: the experience of the Lombardy Region. Health Care Manag Sci.

[13] OECD, Eurostat (2005). Oslo Manual [Internet].

[14] Zulu JM, Hurtig A-K, Kinsman J, Michelo C (2015). Innovation in health service delivery: integrating community health assistants into the health system at district level in Zambia. BMC Health Serv Res.

[15] Abernethy MA, Stoelwinder JU (1995). The role of professional control in the management of complex organizations. Account Organ Soc.

[16] Mahlendorf MD, Kleinschmit F, Perego P (2014). Relational effects of relative performance information: The role of professional identity. Account Organ Soc.

[17] Simons R (1994). How new top managers use control systems as levers of strategic renewal. Strateg Manag J.

[18] Mutiganda JC (2013). Budgetary governance and accountability in public sector organisations: An institutional and critical realism approach. Crit Perspect Account.

[19] Hambrick DC, Abrahamson E (1995). Assessing Managerial Discretion across Industries: A Multimethod Approach. Acad Manage J.

[20] Hambrick DC, Finkelstein S (1987). Managerial discretion: A bridge between polar views of organizational outcomes. Res Organ Behav.

[21] Simons R (1991). Strategic orientation and top management attention to control systems. Strateg Manag J.

[22] Wangrow DB, Schepker DJ, Barker VL (2014). Managerial Discretion An Empirical Review and Focus on Future Research Directions. J Manag..

[23] Regioni. Sanità: Bonaccini su riparto risorse 2016, “obiettivo raggiunto”, [Internet]. Available from: http://www.regioni.it/comunicato-stampa/2016/02/11/sanita-bonaccini-su-riparto-risorse-2016-obiettivo-raggiunto-443526/. Cited 27 July 2016

[24] Bartlett W, Grand JL, Grand JL, Bartlett W (1993). The Theory of Quasi-Markets. Quasi-Markets and Social Policy [Internet].

[25] Fattore G, Torbica A (2006). Inpatient reimbursement system in Italy: How do tariffs relate to costs?. Health Care Manag Sci.

[26] Garavaglia G, Lettieri E, Agasisti T, Lopez S (2010). Efficiency and quality of care in nursing homes: an Italian case study. Health Care Manag Sci.

[27] Brenna E, Spandonaro F (2015). Regional incentives and patient cross-border mobility: evidence from the Italian experience. Int J Health Policy Manag.

[28] Lo Scalzo A, Donatini A, Orzella L, Cicchetti A, Profili S, Maresso A (2009). Italy: Health system review. Health systems in transition.

[29] Nuti S, Seghieri C, Vainieri M (2012). Assessing the effectiveness of a performance evaluation system in the public health care sector: some novel evidence from the Tuscany region experience. J Manag Gov.

[30] Fusco D, Davoli M, Pinnarelli L, Colais P, D’Ovidio M, Basiglini A (2012). Il Programma Nazionale di valutazione Esiti (PNE). Breve guida alla consultazione. Monitor.

[31] Cavalieri M, Gitto L, Guccio C (2013). Reimbursement systems and quality of hospital care: an empirical analysis for Italy. Health Policy Amst Neth.

[32] Macinati MS (2008). The relationship between quality management systems and organizational performance in the Italian National Health Service. Health Policy Amst Neth.

[33] Conforti D, Guerriero F, Guido R, Cerinic MM, Conforti ML (2010). An optimal decision making model for supporting week hospital management. Health Care Manag Sci.

[34] Ozcan YA, Tànfani E, Testi A (2016). Improving the performance of surgery-based clinical pathways: a simulation-optimization approach. Health Care Manag Sci.

[35] Bisbe J, Batista-Foguet J-M, Chenhall R (2007). Defining management accounting constructs: A methodological note on the risks of conceptual misspecification. Account Organ Soc.

[36] Demartini C, Mella P. Beyond feedback control: the interactive use of performance management systems. Implications for process innovation in Italian healthcare organizations. Int J Health Plann Manage. 2014;8:e1–e30. 10.1002/hpm.2177 23564664

[37] Eccles RG, Serafeim G (2013). The Performance Frontier.

[38] Cattinelli I, Bolzoni E, Barbieri C, Mari F, Martin-Guerrero JD, Soria-Olivas E (2011). Use of Self-Organizing Maps for Balanced Scorecard analysis to monitor the performance of dialysis clinic chains. Health Care Manag Sci.

[39] Naranjo-Gil D, Hartmann F (2007). How CEOs use management information systems for strategy implementation in hospitals. Health Policy.

[40] Kacak H, Ozcan YA, Kavuncubasi S (2014). A new examination of hospital performance after healthcare reform in Turkey: sensitivity and quality comparisons. Int J Public Policy.

[41] Ketchen DJ, Palmer TB, Gamm LD (2001). The role of performance referents in health services organizations. Health Care Manage Rev.

[42] Georgescu I, Hartmann FGH (2013). Sources of financial pressure and up coding behavior in French public hospitals. Health Policy.

[43] Gregory S (2015). William Pickles Lecture 2014: Cum Scientia Caritas — compassion with knowledge. Br J Gen Pract.

[44] Carpenter MA, Golden BR (1997). Perceived Managerial Discretion: A Study of Cause and Effect. Strateg Manag J.

[45] Phillips RA, Berman SL, Elms H, Johnson-Cramer ME (2010). Strategy, stakeholders and managerial discretion. Strateg Organ.

[46] Hope O-K, Thomas WB, Vyas D (2013). Financial Reporting Quality of U.S. Private and Public Firms. Account Rev.

[47] Kaplan RS, Norton DP (2001). Transforming the Balanced Scorecard from Performance Measurement to Strategic Management: Part I. Account Horiz.

[48] Zelman WN, Pink GH, Matthias CB (2003). Use of the balanced scorecard in health care. J Health Care Finance.

[49] Naranjo-Gil D, Hartmann F (2007). Management accounting systems, top management team heterogeneity and strategic change. Account Organ Soc.

[50] Artz M, Homburg C, Rajab T (2012). Performance-measurement system design and functional strategic decision influence: The role of performance-measure properties. Account Organ Soc.

[51] Spence Laschinger HK, Shamian J (1994). Staff Nurses’ and Nurse Managers’ Perceptions of Job-Related EMpowerment and Self-Efficacy. J Nurs Adm.

[52] Hoff T (2011). Deskilling and adaptation among primary care physicians using two work innovations. Health Care Manage Rev.

[53] Berwick DM (2003). Disseminating innovations in health care. JAMA.

[54] SISTAN (2010). Annuario Statistico Regionale Lombardia [Internet]. ISTAT.

[55] Dawes M, Sampson U (2003). Knowledge management in clinical practice: a systematic review of information seeking behavior in physicians. Int J Med Inf.

[56] Messiah A, Lacoste J, Gokalsing E, Shultz JM, Rodríguez de la Vega P, Castro G (2016). Mental Health Impact of Hosting Disaster Refugees: Analyses from a Random Sample Survey Among Haitians Living in Miami. South Med J.

[57] Marshall MN (1996). Sampling for qualitative research. Fam Pract.

[58] Armstrong JS, Overton TS. Estimating nonresponse bias in mail surveys. J Mark Res. 1977;396–402.

[59] Sapsford R. Survey Research. London: Sage; 2006. p. 292.

[60] Bartlett J, Kotrlik J, Higgins C (2001). Organizational Research: Determining Appropriate Sample Size in Survey Research. Inf Technol Learn Perform J.

[61] Barclay DW, Higgins C, Thompson R (1995). The partial least squares (PLS) approach to causal modeling: Personal computer adaptation and use as an illustration. Technol Stud.

[62] Dillman DA. Mail and Internet Surveys: The Tailored Design Method -- 2007 Update with New Internet, Visual, and Mixed-Mode Guide. New York: Wiley; 2006. p. 543.

[63] Riedler J, Braun-Fahrländer C, Eder W, Schreuer M, Waser M, Maisch S (2001). Exposure to farming in early life and development of asthma and allergy: a cross-sectional survey. Lancet Lond Engl.

[64] Meredith C, Symonds P, Webster L, Lamont D, Pyper E, Gillis CR (1996). Information needs of cancer patients in west Scotland: cross sectional survey of patients’ views. BMJ.

[65] Ellaway A, Macintyre S, Bonnefoy X (2005). Graffiti, greenery, and obesity in adults: secondary analysis of European cross sectional survey. BMJ.

[66] Abrahams SC, Keve ET (1971). Normal probability plot analysis of error in measured and derived quantities and standard deviations. Acta Crystallogr A.

[67] Lin I-F, Schaeffer NC (1995). Using Survey Participants to Estimate the Impact of Nonparticipation. Public Opin Q.

[68] Voigt LF, Koepsell TD, Daling JR (2003). Characteristics of telephone survey respondents according to willingness to participate. Am J Epidemiol.

[69] Kogut B, Kulatilaka N (1994). Operating Flexibility, Global Manufacturing, and the Option Value of a Multinational Network. Manag Sci.

[70] Govindarajan V (1984). Appropriateness of accounting data in performance evaluation: An empirical examination of environmental uncertainty as an intervening variable. Account Organ Soc.

[71] Ringle CM, Sarstedt M, Straub DW (2012). Editor’s Comments: A Critical Look at the Use of PLS-SEM in MIS Quarterly. MIS Q.

[72] Henseler J, Ringle CM, Sinkovics RR. The use of partial least squares path modeling in international marketing. In: New Challenges to International Marketing [Internet], vol. 20. Emerald Group Publishing Limited; 2009. p. 277–319. Advances in International Marketing). Available from: http://www.emeraldinsight.com/doi/abs/10.1108/S1474-7979(2009)0000020014. Cited 2 Feb 2015.

[73] Hulland J (1999). Use of partial least squares (PLS) in strategic management research: a review of four recent studies. Strateg Manag J.

[74] Macinati MS, Anessi-Pessina E (2014). Management accounting use and financial performance in public health-care organisations: Evidence from the Italian National Health Service. Health Policy.

[75] Chin WW. The Partial Least Squares Approach to Structural Equation Modeling. In: Marcoulides GA, editor. Modern Methods for Business Research. Mahwah: Lawrence Erlbaum; 1998. p. 295–358.

[76] Podsakoff PM (2003). Common method biases in behavioral research: a critical review of the literature and recommended remedies. J Appl Psychol.

[77] Bisbe J, Otley D (2004). The effects of the interactive use of management control systems on product innovation. Account Organ Soc.

[78] Raisch S, Birkinshaw J, Probst G, Tushman ML (2009). Organizational Ambidexterity: Balancing Exploitation and Exploration for Sustained Performance. Organ Sci.

[79] Cho TS, Hambrick DC (2006). Attention as the Mediator Between Top Management Team Characteristics and Strategic Change: The Case of Airline Deregulation. Organ Sci.

[80] Langabeer JR, Yao E (2012). The impact of chief executive officer optimism on hospital strategic decision making. Health Care Manage Rev.

[81] Neriz L, Silva D, Ramis F, Nunez A (2014). Tools to manage the decision-making process in operating rooms. BMC Health Serv Res.

[82] Weisbrod BA (1991). The Health Care Quadrilemma: An Essay on Technological Change, Insurance, Quality of Care, and Cost Containment. J Econ Lit.

[83] Mauro M, Cardamone E, Cavallaro G, Minvielle E, Rania F, Sicotte C (2014). Teaching hospital performance: Towards a community of shared values?. Soc Sci Med.

[84] Epstein L (2014). Data-based decision-making at all managerial levels in health care: an integral part of evidence-based practice. BMC Health Serv Res.

[85] Speiser SM, Malawer SS. American Tragedy: Damages for Mental Anguish of Bereaved Relatives in Wrongful Death Actions. Tulane Law Rev. 1976 1977;51:1.

[86] Rogelberg SG, Stanton JM (2007). Introduction: Understanding and dealing with organizational survey nonresponse. Organ Res Methods.

[87] Armstrong J, Overton T. Estimating Nonresponse Bias in Mail Surveys [Internet]. EconWPA; 2005. Available from: http://econpapers.repec.org/paper/wpawuwpgt/0502044.htm. Cited 2 Nov 2015.

[88] Syam SS, Côté MJ (2010). A location–allocation model for service providers with application to not-for-profit health care organizations. Omega.

[89] Chenhall RH (2003). Management control systems design within its organizational context: findings from contingency-based research and directions for the future. Account Organ Soc.

[90] Bruce N, Pope D, Stanistreet D. Standardisation. In: Quantitative Methods for Health Research [Internet]. John Wiley & Sons, Ltd; 2008. p. 111–28. Available from: http://onlinelibrary.wiley.com/doi/10.1002/9780470725337.ch3/summary. Cited 2 Nov 2015.

[91] Siegel S (1956). Nonparametric statistics for the behavioral sciences.

