# Supplementary material for: Are performance measurement systems useful? Perceptions from health care
Source: BMC Health Serv Res. 2017 Jan 31;17:96. doi: 10.1186/s12913-017-2022-9 (PMC5282789; doi:10.1186/s12913-017-2022-9)

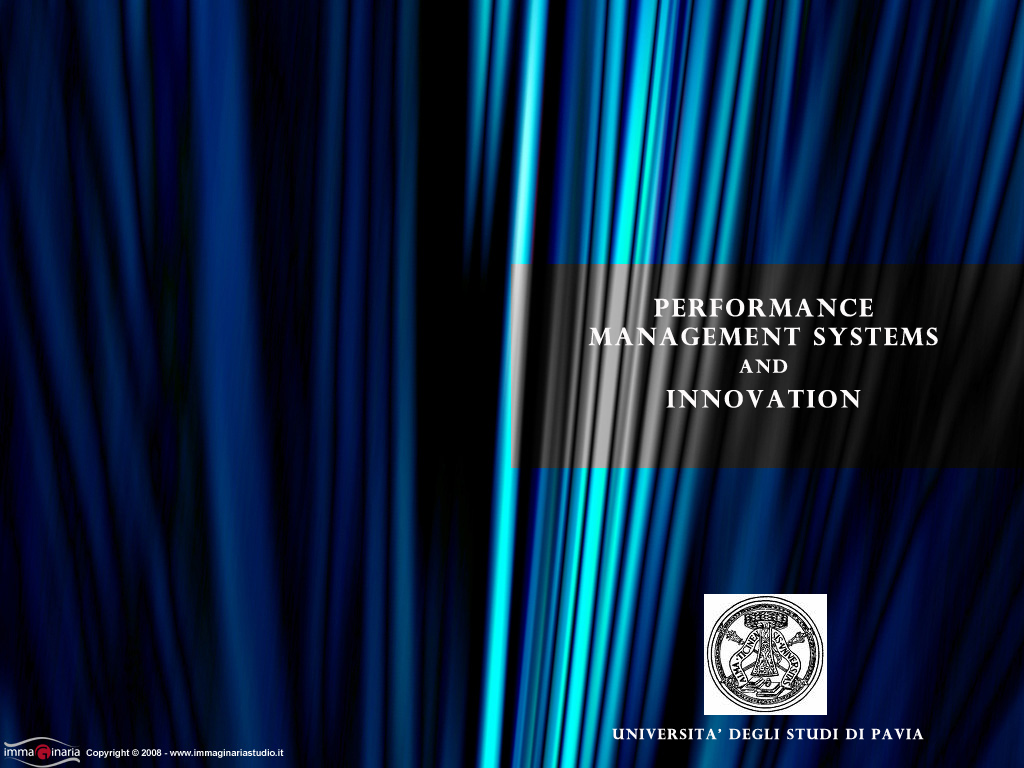

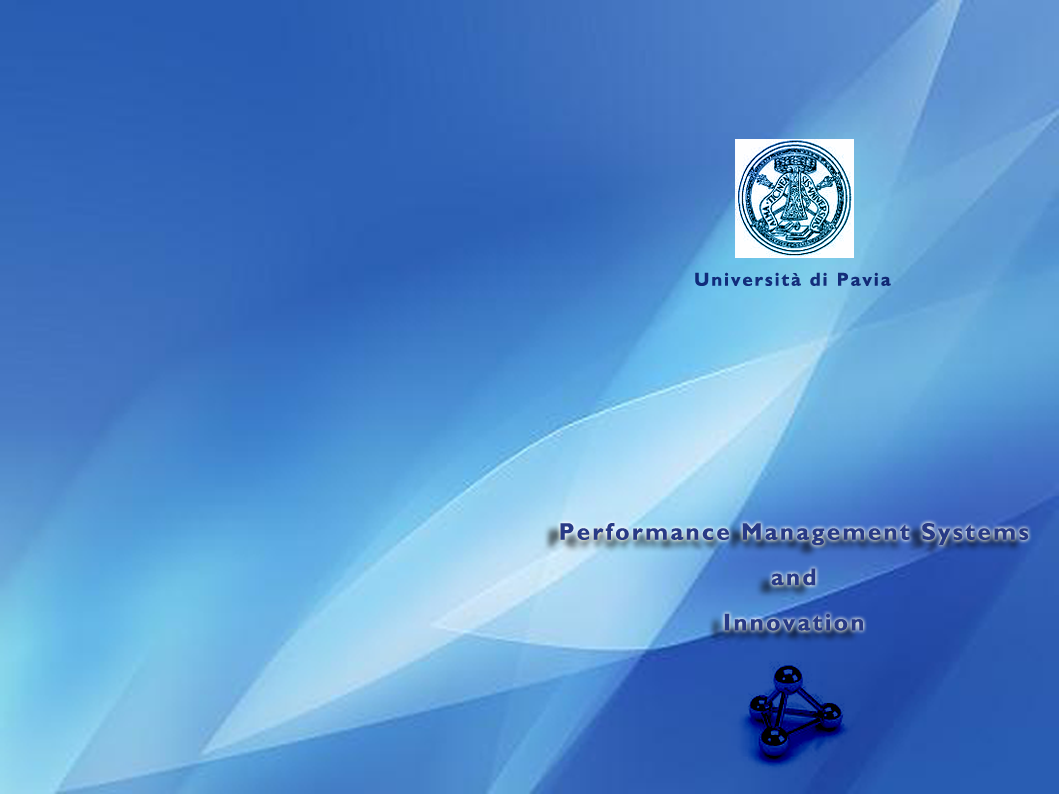


**Performance Management System
and
Innovation**

**Section 1. PERFORMANCE MANAGEMENT SYSTEM EFFECTIVENESS**

| *A* **non-financial performance system** *is a set of non-financial based performance measures that identify key drivers to organisational success. A typical example of non-financial performance systems is the Balanced Scorecard (excluding the financial perspective).* |
| --- |

1. How would you rate the effectiveness of the **non-financial performance system** used within your unit in providing information with reference to the following aspects?

|  | **Extremely unsatisfactory** | | **Neither satisfactory nor unsatisfactory** | | | | **Extremely satisfactory** | |
| --- | --- | --- | --- | --- | --- | --- | --- | --- |
| To support operational decisions of your unit | 1 | 2 | 3 | 4 | 5 | 6 | | 7 |
| To enable flexibility/ adaptability of your unit | 1 | 2 | 3 | 4 | 5 | 6 | | 7 |

2. How would you rate your overall satisfaction with the following mechanisms in use within your unit?

|  | **Extremely unsatisfactory** | | **Neither satisfactory nor unsatisfactory** | | | | **Extremely satisfactory** | |
| --- | --- | --- | --- | --- | --- | --- | --- | --- |
| Non-financial performance system | 1 | 2 | 3 | 4 | 5 | 6 | | 7 |

Strategic uncertainties *relates to changes in competitive dynamics and internal competencies that may create opportunities or threats*

3. How would you rate the use of the following performance management system mechanisms in **detecting strategic uncertainties** within your unit?

|  | **Extremely unsatisfactory** | | **Neither satisfactory nor unsatisfactory** | | | | **Extremely satisfactory** | |
| --- | --- | --- | --- | --- | --- | --- | --- | --- |
| Non-financial performance system | 1 | 2 | 3 | 4 | 5 | 6 | | 7 |

**Section 2. PROCESS INNOVATION**

*Process innovations are all new or significantly improved methods for the production or supply of products (goods and services). The innovation although new to the business, does not need to be new to your sector.*

4. During the last three years did your unit undertake any **process innovations**?

Yes

No **If NO, go to question 6.**

5. During the last three years, your unit introduced

|  | | **Very much less than sector’s average** | | **Same as  sector’s average** | | | | **Much greater than sector’s average** | |
| --- | --- | --- | --- | --- | --- | --- | --- | --- | --- |
| Significantly improved processes | 1 | | 2 | 3 | 4 | 5 | 6 | | 7 |

**Section 3. CONTEXTUAL VARIABLES**

6. How would you rate the following environment related aspects faced in your unit’s environment compared to the average of the sector you belong to?

|  | | **Very much less than sector’s average** | | **Same as  sector’s average** | | | | **Much greater than sector’s average** | |
| --- | --- | --- | --- | --- | --- | --- | --- | --- | --- |
| Degree of Uncertainty | 1 | | 2 | 3 | 4 | 5 | 6 | | 7 |
| Level of Risk | 1 | | 2 | 3 | 4 | 5 | 6 | | 7 |
| Level of Complexity | 1 | | 2 | 3 | 4 | 5 | 6 | | 7 |
| Amount of Change | 1 | | 2 | 3 | 4 | 5 | 6 | | 7 |

**Section 4. DEMOGRAPHIC INFORMATION**

7.a Gender

- Female
- Male

7.b How long have you been with the company?

…………………………….(*years*) …………………………….(*months*)

7.c How long have you been in your current job?

…………………………….(*years*) …………………………….(*months*)

**Please provide any comments**

If there is anything you would like to tell us about this survey please do so in the space provided below or attach a separate sheet of paper.

|  |
| --- |

**Thank you for your contribution to this study!**

**Please return the completed questionnaire in the enclosed envelop to**

**Chiara Demartini**

**Facoltà di Economia**

**Via S. Felice, 5/7**

**27100 Pavia (PV)**

**Italy**


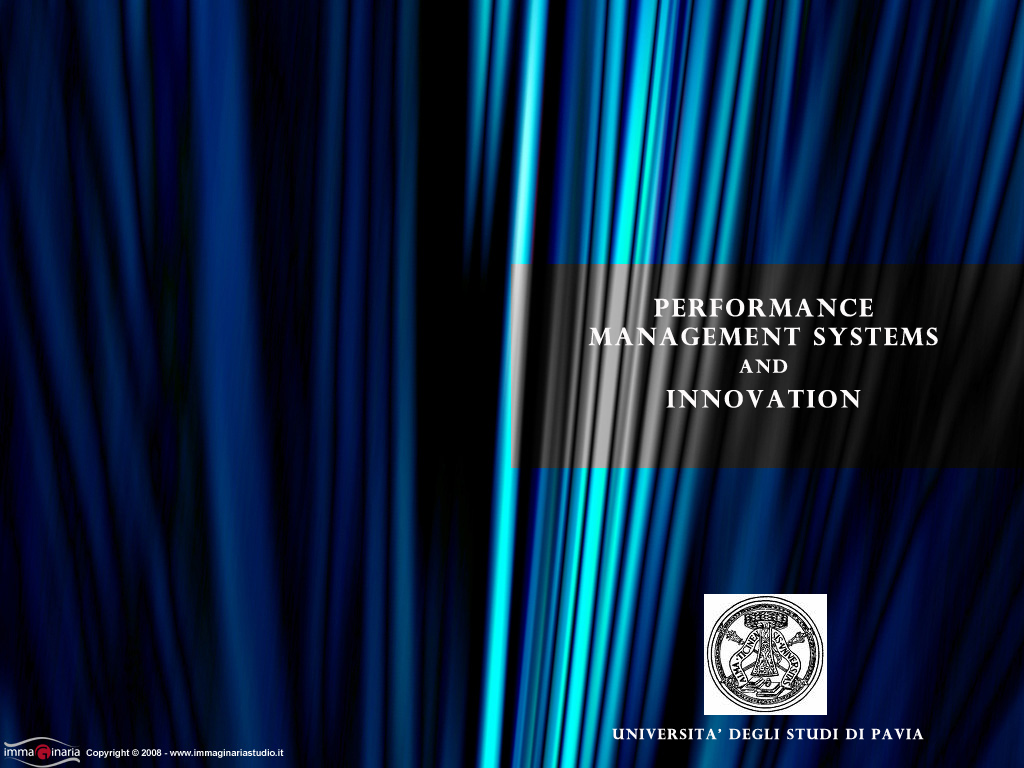

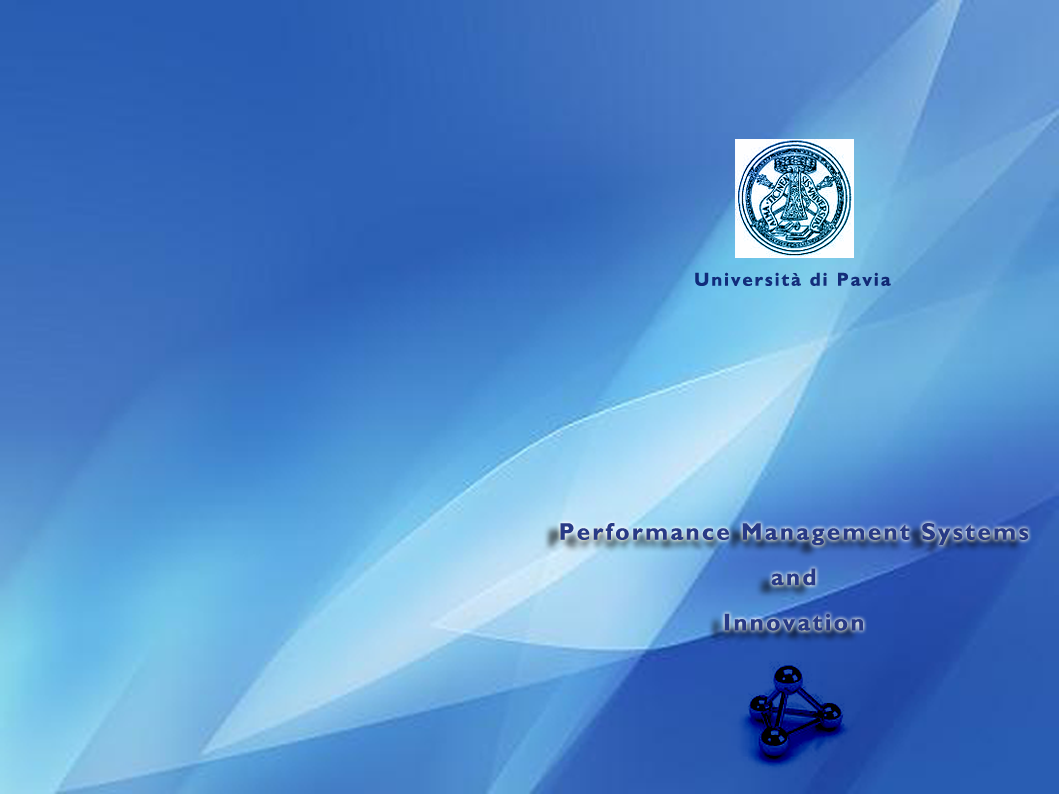

Supplement: Additional file 1: — Questionnaire. This file provides information regarding the questions included into the survey related to this study. (DOCX 1713 kb) [file 12913_2017_2022_MOESM1_ESM.docx]
